# External validation of a prehospital risk score for critical illness

**DOI:** 10.1186/s13054-016-1408-0

**Published:** 2016-08-11

**Authors:** Daniel R. Kievlan, Christian Martin-Gill, Jeremy M. Kahn, Clifton W. Callaway, Donald M. Yealy, Derek C. Angus, Christopher W. Seymour

**Affiliations:** 1 Department of Critical Care Medicine, University of Pittsburgh School of Medicine, 3550 Terrace Street, Scaife Hall #607, Pittsburgh, PA 15261 USA; 2 Clinical Research, Investigation, and Systems Modeling of Acute Illness (CRISMA) Center, Pittsburgh, PA USA; 3 Department of Emergency Medicine, University of Pittsburgh, Pittsburgh, PA, USA

**Keywords:** Clinical decision support systems, Critical illness, Emergency medical services, Forecasting, Prognosis, Triage

## Abstract

**Background:**

Identification of critically ill patients during prehospital care could facilitate early treatment and aid in the regionalization of critical care. Tools to consistently identify those in the field with or at higher risk of developing critical illness do not exist. We sought to validate a prehospital critical illness risk score that uses objective clinical variables in a contemporary cohort of geographically and temporally distinct prehospital encounters.

**Methods:**

We linked prehospital encounters at 21 emergency medical services (EMS) agencies to inpatient electronic health records at nine hospitals in southwestern Pennsylvania from 2010 to 2012. The primary outcome was critical illness during hospitalization, defined as an intensive care unit stay with delivery of organ support (mechanical ventilation or vasopressor use). We calculated the prehospital risk score using demographics and first vital signs from eligible EMS encounters, and we tested the association between score variables and critical illness using multivariable logistic regression. Discrimination was assessed using the AUROC curve, and calibration was determined by plotting observed versus expected events across score values. Operating characteristics were calculated at score thresholds.

**Results:**

Among 42,550 nontrauma, non-cardiac arrest adult EMS patients, 1926 (4.5 %) developed critical illness during hospitalization. We observed moderate discrimination of the prehospital critical illness risk score (AUROC 0.73, 95 % CI 0.72–0.74) and adequate calibration based on observed versus expected plots. At a score threshold of 2, sensitivity was 0.63 (95 % CI 0.61–0.75), specificity was 0.73 (95 % CI 0.72–0.73), negative predictive value was 0.98 (95 % CI 0.98–0.98), and positive predictive value was 0.10 (95 % CI 0.09–0.10). The risk score performance was greater with alternative definitions of critical illness, including in-hospital mortality (AUROC 0.77, 95 % CI 0.7 –0.78).

**Conclusions:**

In an external validation cohort, a prehospital risk score using objective clinical data had moderate discrimination for critical illness during hospitalization.

**Electronic supplementary material:**

The online version of this article (doi:10.1186/s13054-016-1408-0) contains supplementary material, which is available to authorized users.

## Background

Emergency medical services (EMS) agencies transport over 28 million patients per year in the United States [1]. Many of these patients have critical illness and experience substantial morbidity and mortality during subsequent hospitalization. The recognition of critical illness during prehospital care by EMS could lead to redistribution of patients to regional centers of excellence or prompt specific treatment before hospital arrival [2–5]. These strategies better match patient needs with critical care resources and are used in many time-sensitive conditions such as traumatic injury, acute cardiovascular disease, and cardiac arrest [6, 7].

Yet, the recognition of high-risk prehospital patients is challenging for clinicians. In the brief prehospital time interval, paramedics’ subjective assessments may not adequately discriminate patients who require hospital admission [8], and combinations with objective data offer only modest improvement [9]. Another approach is to use only objective prehospital data in risk assessments, but these may be missing or perform poorly as single measurements [10].

In prior work, a prehospital critical illness risk model used multiple objective, commonly recorded variables and adequately predicted the development of critical illness during hospitalization in a regional EMS system [11]. Although internally validated and tested in the emergency department [12], this model has yet to be externally validated using temporally and geographically distinct EMS data. We sought to validate model performance in a contemporary cohort of 21 EMS agencies transporting to 9 hospitals in an integrated healthcare system.

## Methods

### Study design, population, and setting

The institutional review board of the University of Pittsburgh approved the study with a waiver of informed consent. Following the Transparent Reporting of a multivariable prediction model for Individual Prognosis or Diagnosis (TRIPOD) recommendations for external validation of clinical risk scores [13–15], we linked EMS encounters from 21 agencies to inpatient electronic health records (EHRs) at 9 hospitals of the UPMC health system from January 2010 to December 2012. All EMS agencies received medical command in a two-tier system through the UPMC Department of Emergency Medicine, with on-scene medical care primarily provided by paramedics trained in advanced life support. Standardized prehospital electronic records of these encounters are stored in a secure repository (*emsCharts*, Warrendale, PA, USA), and they were linked to hospital EHR data using hierarchical matching (Cerner PowerChart; Cerner Corporation, North Kansas City, MO, USA) as previously described [16]. We included only scene-to-hospital transports of adult patients ≥18 years of age. We excluded transports for cardiac arrest, trauma, burn, or falls or EMS records that lacked adequate clinical documentation to determine the prehospital risk score. We also excluded duplicate encounters and data from three geographically distinct hospitals that each received fewer than ten EMS transports from participating agencies.

### Variable definitions

We defined the primary outcome of critical illness during hospitalization as intensive care unit (ICU) location stated in the EHRs with concomitant delivery of organ support (either mechanical ventilation or vasopressor use). The delivery of mechanical ventilation was identified using intubation, extubation, and tracheostomy events and ventilator mode data in the EHRs. Vasopressor use was defined as the administration of vasoactive agents (e.g., norepinephrine, dopamine, epinephrine) by infusion for more than 1 h recorded in the EHRs.

### Model assessment and data analysis

We determined the prehospital risk score among eligible EMS encounters using demographics and the initial prehospital vital signs. Risk score variables, including age, sex, respiratory rate, systolic blood pressure, heart rate, pulse oximetry, and Glasgow Coma Scale (GCS) score, were categorized according to prior thresholds [11]. We assigned integer points for each category as previously reported (Additional file 1: Table S2) and summed the points to determine the total score (range 0–8) for each EMS encounter. When a necessary variable was missing, we used single-value imputation, assuming normal, as is standard in most critical illness scores [17, 18]. We used Pearson’s chi-square test with *p* < 0.05 to assess for a difference in distributions of score values among encounters in which critical illness developed. We assessed model discrimination using the AUROC curve with binomial CIs. Because calibration statistics such as the Hosmer-Lemeshow statistic are often statistically significant in large datasets [19], we evaluated model calibration by graphically assessing a plot of observed versus expected events across the score range. We calculated sensitivity, specificity, and positive and negative predictive values for clinically relevant score thresholds.

### Sensitivity analyses

We performed several sensitivity analyses to assess the robustness of our findings. We explored model performance for alternative definitions of critical illness: (1) the critical illness outcome measured in the primary publication (any one of severe sepsis using the Angus implementation of International Classification of Diseases, Ninth Revision, Clinical Modification [ICD-9-CM], codes [20]; mechanical ventilation ≥72 h from ICD-9-CM procedure codes; or in-hospital mortality) [11], (2) an EHR definition using only organ support (either receipt of mechanical ventilation or vasopressor use), and (3) an EHR definition using only in-hospital mortality. We also determined score performance in the following a priori analyses: (1) use of worst vital signs rather than initial vital signs, as these may more accurately reflect patient deterioration; (2) a restricted cohort of patient encounters with transport times greater than the median, as this could inform generalizability to rural EMS systems [21]; and (3) exclusion of patients with Do Not Intubate orders, as these patients may be misclassified by outcome definitions that use mechanical ventilation. Finally, we reweighted the categorized score by rounding beta coefficients of the external validation multivariable logistic regression model to the nearest integers. We then determined if model performance was improved using these reweighted point values [15]. Among sensitivity analyses, we considered a chi-square test result of the AUROC area with *p* < 0.05 to indicate a statistically significant difference in performance compared with the main risk score. All analyses were performed with STATA 13.0 software (StataCorp, College Station, TX, USA). All tests of significance used a two-sided *p* ≤ 0.05.

## Results

**Fig. 1:**
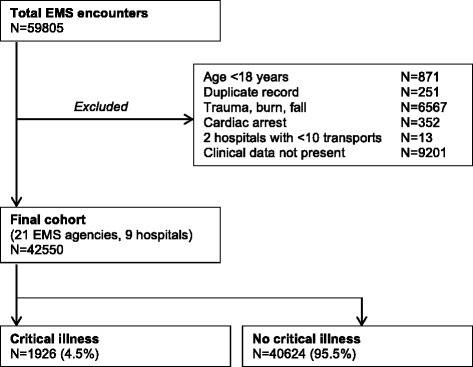
Patient accrual. *EMS* emergency medical services

Among 59,805 prehospital encounters (Fig. 1), we excluded those less than 18 years of age (*n* = 871, 1.5 %); those with trauma, burn, or fall (*n* = 6567, 11.0 %); those with cardiac arrest (*n* = 352, 0.6 %); and those for whom prehospital risk score data were not available (*n* = 9201, 15.4 %). The final cohort consisted of 42,550 encounters in which 1926 patients (4.5 %) developed critical illness during their hospitalization according to the primary definition. Compared with encounters in which patients did not develop critical illness, critically ill patients were older, more frequently male, and more likely to present with prehospital respiratory or neurological symptoms (*p* < 0.01 for all) (Table 1). Encounters that developed critical illness were also more likely to receive supplemental oxygen, have peripheral intravenous access established, and undergo endotracheal intubation prior to hospital arrival (*p* < 0.01). Hospital length of stay and in-hospital mortality were greater among critically ill patients (*p* < 0.01).

**Table 1:** Patient characteristics

Variable	Critical illness	No critical illness	*p* Value
Number of patients (%)	1926 (4.5)	40,624 (95.5)	
Age, years, mean (SD)	66 (17)	62 (21)	<0.01
Male sex, n (%)	929 (48)	15,987 (39)	<0.01
EMS staffing level, n (%)			<0.01
Critical care	188 (10)	427 (1)	
ALS	1676 (87)	37,646 (93)	
BLS	61 (3)	2540 (6)	
Initial prehospital vital signs, mean ± SD			
Systolic blood pressure, mmHg	131 ± 36	139 ± 28	<0.01
Heart rate, beats/minute	96 ± 26	89 ± 20	<0.01
Respiratory rate, breaths/minute	20 ± 7	19 ± 5	<0.01
Oxygen saturation, %	93 ± 9	96 ± 10	<0.01
Glasgow Coma Scale score	12 ± 4	14 ± 2	<0.01
Diagnostic category, *n* (%)^a^			<0.01
Respiratory	405 (21)	4863 (12)	
Neurological	475 (25)	6733 (17)	
Cardiovascular	180 (9)	4199 (10)	
Abdominal	134 (7)	4208 (10)	
Metabolic/endocrine	22 (1)	683 (2)	
Psychiatric/toxicology	52 (3)	1000 (2)	
Fall	94 (5)	4310 (11)	
Obstetric/gynecologic	1 (<1)	374 (1)	
Medical (NOS)	386 (20)	8025 (20)	
Other	138 (7)	2356 (6)	
Scene to hospital destination, miles, median [IQR]	6.5 [3.5–9.3]	5.8 [2.8–8.0]	<0.01
Prehospital interventions, n (%)			
Intubation	128 (7)	86 (<1)	<0.01
Supplemental oxygen	1001 (58)	14,626 (44)	<0.01
ECG monitoring	340 (20)	5807 (17)	<0.02
Peripheral or central intravenous access	1135 (66)	19,160 (57)	<0.01
Components of critical illness, n (%)			
Ever received mechanical ventilation	1643 (85)	208 (<1)	<0.01
Ever received vasopressor	912 (47)	65 (<1)	<0.01
ICU location in electronic health record	1926 (100)	3279 (8)	<0.01
Hospital length of stay, days, median [IQR]	10 [6–17]	2 [1–5]	<0.01
Hospital mortality, n (%)	463 (24)	447 (1)	<0.01

*Abbreviations: EMS* emergency medical services, *ALS* advanced life support, *BLS* basic life support, *NOS* not otherwise specified, *ECG* electrocardiogram, *ICU* intensive care unit, *IQR* interquartile range

^a^Diagnostic category was determined by EMS staff impression

**Fig. 2:**
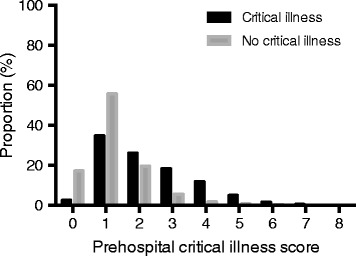
Distribution of prehospital risk scores of patients with critical illness (*black bars*) versus those without critical illness (*gray bars*)

**Fig. 3:**
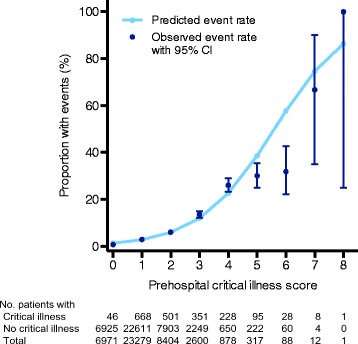
Calibration curve showing the expected rate of critical illness compared with the observed rate (with 95 % CI) for each risk score value

A total of 71.1 % of encounters (*n* = 30,250) had a prehospital risk score of 0 to 1, while 25.9 % (*n* = 11,004) had a score of 2 or 3 and 3.0 % (*n* = 1296) had a score >3. The proportion of patients receiving mechanical ventilation, vasopressors, and intensive care increased with greater prehospital risk scores (Additional file 1: Figure S1). When stratified by critical illness, the prehospital risk scores were higher among the critically ill (Fig. 2) (*p* < 0.01 by Pearson’s chi-square test). The prehospital risk score demonstrated satisfactory discrimination for critical illness (AUROC 0.73, 95 % CI 0.72–0.74). Calibration of the risk score was adequate on the basis of observed versus expected plots (Fig. 3). Using a threshold score ≥1 to identify critical illness, we observed a sensitivity of 0.98 (95 % CI 0.97–0.98), specificity of 0.17 (95 % CI 0.17–0.17), positive predictive value of 0.05 (95 % CI 0.05–0.06), and negative predictive value of 0.99 (95 % CI 0.99–1.0). Using a score threshold ≥2 to identify critical illness, sensitivity decreased with little change in positive or negative predictive value (Table 2).

**Table 2:** Operating characteristics for prehospital risk score thresholds

	Prehospital risk score threshold		
Operating characteristics	1	2	3
Sensitivity, 95 % CI	97.6 (96.8–98.3)	62.9 (60.7–65.1)	36.9 (34.8–39.1)
Specificity, 95 % CI	17.1 (16.7–17.4)	72.7 (72.3–73.1)	92.2 (91.9–92.4)
Negative predictive value, 95 % CI	99.3 (99.1–99.5)	97.6 (97.5–97.8)	96.9 (96.7–97.0)
Positive predictive value, 95 % CI	5.3 (5.1–5.5)	9.9 (9.3–10.4)	18.3 (17.1–19.5)

In sensitivity analyses, the AUROC values for models using alternate definitions of critical illness were similar to those in the primary model (Table 3). Prehospital risk score performance was better when worst prehospital vital signs were used (*p* < 0.01), and correlation between first and worst vital signs was high (Pearson correlation coefficient range 0.69–0.91) (Additional file 1: Table S1). Score performance was similar in cohorts restricted to longer than median transport times or those without limitations to life-sustaining therapy. When risk score variables were reweighted using multivariable logistic regression, point scores were different within strata of respiratory rate and GCS score, resulting in a model with range from 0 to 7 points (Additional file 1: Table S2). In the revised model, GCS <8 had the greatest weight (integer score of 2 points), compared with the original model in which this stratum and respiratory rate ≥36 shared the greatest weight (integer score of 2 points). The revised model had discrimination for critical illness similar to that of the primary model (*p* = 0.77).

**Table 3:** Discrimination of the prehospital risk score in the primary model, alternative definitions of critical illness, and sensitivity analyses

	AUROC	95 % CI
Primary model	0.73	0.72–0.74
Alternative definitions of critical illness		
In-hospital mortality	0.77	0.76–0.79
Any organ support event	0.72	0.71–0.73
Administrative claims	0.71	0.70–0.72
Sensitivity analyses		
Worst vital signs	0.74	0.73–0.75
Longer than median transport distance	0.71	0.69–0.72
Excluding patients with Do Not Intubate orders	0.73	0.72–0.75
Model with reweighting of coefficients	0.73	0.72–0.74

## Discussion

We externally validated a prehospital risk score that predicts critical illness during hospitalization in a multiagency regional EMS system. The score uses objective variables, including demographics and vital signs, that are commonly recorded during prehospital care to discriminate patients who will develop critical illness. These data help advance efforts to identify non-cardiac arrest, nontrauma patients at greatest risk of critical illness during very early care, an opportunity for rapid risk assessment that may inform direct treatment or triage to centers of excellence.

Researchers in many observational studies have proposed that patients with respiratory failure requiring mechanical ventilation [2], sepsis [7], or critical illness have improved outcomes at higher-volume centers [22]. Critical illness regionalization is often suggested as a strategy to leverage these relationships into higher-quality, more efficient care [23]. A primary barrier to efficient regionalization is the absence of validated tools to guide patient triage with critical illness. Our work addresses this knowledge gap by validating a tool for critical illness prehospital triage. Both the overall discrimination and prehospital physiology were similar when we compared the external cohort with the original cohort [11]. Of note, there are strategies for regionalized care that use condition-specific risk assessments such as the 12-lead electrocardiogram and the Los Angeles Prehospital Stroke Screen or the Cincinnati Prehospital Stroke Scale. This prehospital risk score complements these condition-specific tools by functioning as a “score for all” among a heterogeneous group of prehospital encounters, and it could be considered for prospective validation. Additional barriers limit regionalization demonstration projects, including uncertainty over which regions to centralize, lack of stakeholder consensus, and the potential impact of new referral patterns on the financial stability of hospitals and healthcare systems [24]. These challenges will be more feasible to address with a validated triage tool, and careful study of stakeholders’ perspectives on patient referrals and the financial effects of regionalization will be necessary prior to and during any demonstration projects.

From a clinical perspective, a prehospital risk score should be both valid and easy to measure. In this study, we assessed the validity of the risk score, but prospective studies of implementation will reveal its timeliness and measurement burden. Because the risk score uses objective, physiologic values routinely recorded during the EMS encounter, it is possible that automated measurement will be feasible, even on mobile devices. The integration of EHRs during EMS care has expanded during the past decade, such that risk models can be determined in a timely fashion and shared with receiving hospitals. Finally, the prehospital risk model provides a foundation upon which potential treatments for the noninjured, non-cardiac arrest patient may be built. Similar to care stratification for prehospital treatment in trauma [25], the prehospital risk score could be used to enrich trials of prehospital interventions for specific risk subgroups.

From a research perspective, the prehospital risk score could be used to standardize and compare otherwise heterogeneous EMS populations. Similarly to risk assessments among hospitalized patients with the Acute Physiology and Chronic Health Evaluation or Logistic Organ Dysfunction System score [17, 26], the prehospital risk score could estimate illness severity during the prehospital phase. These measurements could inform risk adjustment when testing the effectiveness of prehospital interventions on outcome [4]. For quality improvement, the score could identify sentinel, high-risk patients in whom to audit performance, as is used in cardiac arrest, ventilator-associated events, and surgical site infections [27]. The broad applicability of the risk score for these purposes is plausible, as the component variables are data fields already present in the National Emergency Medical Services Information System [28].

We recognize several limitations to our study. There is no gold standard definition for critical illness, so we selected a composite outcome of ICU location in EHRs accompanied by concurrent organ support. Alternative approaches to defining critical illness did not reveal changes in model performance. The risk score’s performance could also have been impacted by cohort selection of patients only transported to UPMC hospitals. In general, the cohort characteristics are similar to other large EMS populations in urban, rural, and semirural regions [11]. To favor parsimony and ease of use, we did not seek to maximize model performance by adding variables. We acknowledge that more complex prediction models (e.g., scores with noninteger point values, classification and regression tree analysis) might improve discrimination and calibration, but at the cost of potentially increasing measurement burden. Finally, the organization of other EMS systems may differ from that of this southwestern Pennsylvania cohort. Because the prehospital critical illness score does not involve variables dependent on EMS care or level of training, these differences should have low impact on the external validity of the results.

## Conclusions

In an external validation cohort, a prehospital risk score using objective clinical data had moderate discrimination for critical illness during hospitalization. Although prospective studies and implementation evaluation are required, these data advance support for the use of simple clinical data to triage risk among prehospital, non-cardiac arrest, nontrauma patients.

## Abbreviations

ALS, advanced life support; APACHE, Acute Physiology and Chronic Health Evaluation; BLS, basic life support; DNI, Do Not Intubate; ECG, electrocardiogram; EHR, electronic health record; EMS, emergency medical services; GCS, Glasgow Coma Scale; ICD-9-CM, International Classification of Diseases, Ninth Revision, Clinical Modification; ICU, intensive care unit; IQR, interquartile range; NOS, not otherwise specified; UPMC, University of Pittsburgh Medical Center

## Additional file

Additional file 1:
**Figure S1.** Proportion of patients across the range of prehospital risk scores with various healthcare use. **Table S1.** Proportion of patients with abnormal first versus worst vital signs within variable strata, and correlation between first and worst vital signs for each variable. **Table S2.** Multivariable logistic regression model output of prehospital risk score variables with critical illness showing reweighting in sensitivity analysis. (DOCX 76 kb)
