# Supplementary material for: External validation of a prehospital risk score for critical illness
Source: Crit Care. 2016 Aug 11;20:255. doi: 10.1186/s13054-016-1408-0 (PMC5050704; doi:10.1186/s13054-016-1408-0)
Supplement: Additional file 1: — Figure S1. Proportion of patients across the range of prehospital risk scores with various healthcare use. Table S1. Proportion of patients with abnormal first versus worst vital signs within variable strata, and correlation between first and worst vital signs for each variable. Table S2. Multivariable logistic regression model output of prehospital risk score variables with critical illness showing reweighting in sensitivity analysis. (DOCX 76 kb) [file 13054_2016_1408_MOESM1_ESM.docx]

**Supplementary Content**

**Figure S1.** Proportion of patients across the range of prehospital risk scores with various healthcare use

**Table S1.** Proportion of patients with abnormal first versus worst vital signs within variable strata, and correlation between first and worst vital signs for each variable

**Table S2.** Multivariable logistic regression model output of prehospital risk score variables with critical illness showing reweighting in sensitivity analysis

**Figure S1.**

**Table S1.** Proportion of patients with abnormal first versus worst vital signs within variable strata and overall correlation between first and worst vital signs for each variable

|  |  | **Total no. (%)** | |  |
| --- | --- | --- | --- | --- |
| **Prehospital risk score variable** | | **First vital signs^a^** | **Worst vital signs** | **Correlation coefficient^c^** |
| Male gender | | 16916 (40) | . | . |
| Age, years | |  |  | . |
|  | <45 | 9637 (23) | . |  |
|  | 45 to 64 | 11967 (28) | . |  |
|  | ≥65 | 20946 (49) | . |  |
|  | missing | 0 (0) | . |  |
| Systolic blood pressure, mmHg | |  |  | 0.89 |
|  | ≤90 | 1448 (3) | 2065 (5) |  |
|  | 91 to 140 | 23336 (55) | 26885 (63) |  |
|  | 141 to 180 | 14011 (33) | 11373 (27) |  |
|  | >180 | 3068 (7) | 1540 (4) |  |
|  | missing | 687 (2) | 687 (2) |  |
| Heart rate, beats per minute | |  |  | 0.91 |
|  | ≤60 | 2467 (6) | 1444 (3) |  |
|  | 61 to 99 | 27827 (65) | 27421 (64) |  |
|  | 100 to 119 | 8251 (19) | 8967 (21) |  |
|  | ≥120 | 3512 (8) | 4225 (10) |  |
|  | missing | 493 (1) | 493 (1) |  |
| Respiratory rate, breaths per minute | |  |  | 0.82 |
|  | <12 | 307 (1) | 72 (0) |  |
|  | 12 to 23 | 36643 (86) | 36474 (86) |  |
|  | 24 to 35 | 4292 (10) | 4583 (11) |  |
|  | ≥36 | 426 (1) | 539 (1) |  |
|  | missing | 882 | 882 (1) |  |
| Oxygen saturation, % | |  |  | 0.69 |
|  | ≥93 | 35408 (83) | 34981 (82) |  |
|  | 88 to 92 | 3333 (8) | 3552 (8) |  |
|  | 80 to 87 | 1208 (3) | 1290 (3) |  |
|  | <80 | 618 (1) | 744 (2) |  |
|  | missing | 1983 (5) | 1983 (5) |  |
| Glasgow Coma Scale score | |  |  | 0.87 |
|  | 15 | 28885 (68) | 28269 (66) |  |
|  | 12 to 14 | 4353 (10) | 4588 (11) |  |
|  | 8 to 11 | 1415 (3) | 1428 (3) |  |
|  | <8 | 1343 (3) | 1711 (4) |  |
|  | missing | 6554 (15) | 6554 (15) |  |
| Nursing home location^b^ | | . | . | . |
| ^a^Data shown is either first vital sign or single measurement for gender and nursing home location | | | | |
| ^b^Nursing home location data not available in this dataset | | | | |
| ^c^Correlation between first and worst vitial signs assessed by Pearson's coefficient using variables on a continuous distribution | | | | |

**Table S2.** Multivariable logistic regression model output of prehospital risk score variables with outcome in re-weighting sensitivity analysis

| **Model variable** | | **Seymour et al., *JAMA* 2010** | |  | **External validation cohort** | |
| --- | --- | --- | --- | --- | --- | --- |
|  | | **Regression coefficient,**  ***B* (95% CI)** | **Original point scores** |  | **Regression coefficient,**  ***B* (95% CI)** | **Points after re-weighting** |
| Male gender | | 0.22 (0.15, 0.25) | 0 |  | 0.35 (0.25, 0.45) | 0 |
| Age, years | |  |  |  |  |  |
|  | <45 | ref | 0 |  | ref | 0 |
|  | 45 to 64 | 0.91 (0.80, 1.02) | 1 |  | 1.10 (0.93, 1.28) | 1 |
|  | ≥65 | 1.32 (1.22, 1.43) | 1 |  | 1.07 (0.89, 1.24) | 1 |
| Systolic blood pressure, mmHg | |  |  |  |  |  |
|  | ≤90 | 0.92 (0.82, 1.0) | 1 |  | 0.86 (0.66, 1.05) | 1 |
|  | 91 to 140 | ref | 0 |  | ref | 0 |
|  | 141 to 180 | -0.37 (-0.45, -0.30) | 0 |  | -0.28 (-0.40, -0.16) | 0 |
|  | >180 | -0.11 (-0.22, -0.01) | 0 |  | -0.09 (-0.27, 0.10) | 0 |
| Heart rate, beats per minute | |  |  |  |  |  |
|  | ≤60 | 0.09 (-0.03, 0.21) | 0 |  | 0.08 (-0.13, 0.30) | 0 |
|  | 61 to 99 | ref | 0 |  | ref | 0 |
|  | 100 to 119 | 0.44 (0.36, 0.52) | 0 |  | 0.36 (0.24, 0.49) | 0 |
|  | ≥120 | 0.77 (0.68, 0.85) | 1 |  | 0.77 (0.62, 0.93) | 1 |
| Respiratory rate, breaths per minute | |  |  |  |  |  |
|  | <12 | 1.35 (1.22, 1.43) | 1 |  | -0.48 (-1.52, 0.56) | 0 |
|  | 12 to 23 | ref | 0 |  | ref | 0 |
|  | 24 to 35 | 0.79 (0.72, 0.86) | 1 |  | 0.64 (0.50, 0.78) | 1 |
|  | ≥36 | 1.54 (1.43, 1.64) | 2 |  | 1.05 (0.77, 1.32) | 1 |
| Oxygen saturation, % | |  |  |  |  |  |
|  | ≥93 | ref | 0 |  | ref | 0 |
|  | 88 to 92 | 0.43 (0.24, 0.61) | 0 |  | 0.34 (0.19, 0.50) | 0 |
|  | 80 to 87 | 0.83 (0.61, 1.04) | 1 |  | 0.64 (0.43, 0.85) | 1 |
|  | <80 | 1.08 (0.82, 1.35) | 1 |  | 0.58 (0.31, 0.85) | 1 |
| Glasgow Coma Scale score | |  |  |  |  |  |
|  | 15 | ref |  |  | ref |  |
|  | 12 to 14 | 0.51 (0.38, 0.63) | 1 |  | 0.35 (0.19, 0.51) | 0 |
|  | 8 to 11 | 1.24 (1.10, 1.39) | 1 |  | 1.28 (1.09, 1.46) | 1 |
|  | <8 | 1.96 (1.81, 2.10) | 2 |  | 2.21 (2.05, 2.37) | 2 |
| Nursing home location | | 0.46 (0.36, 0.54) | 0 |  | Not available | 0 |
